# Panton-Valentine Leukocidin in Necrotizing Pneumonia: A Case Report

**DOI:** 10.7759/cureus.78774

**Published:** 2025-02-09

**Authors:** Carla A Costa, Hugo Inácio, Sara Trevas, Ângela Simas

**Affiliations:** 1 Department of Intensive Care Medicine, Unidade Local de Saúde Loures Odivelas, Loures, PRT

**Keywords:** community-acquired pneumonia, intravenous immunoglobulin, necrotizing pneumonia, panton-valentine leucocidin, staphylococcus aureus

## Abstract

Panton-Valentine leukocidin (PVL)-producing *Staphylococcus aureus (S. aureus)* is a rare but clinically significant cause of community-acquired pneumonia (CAP). This toxin leads to extensive tissue destruction and severe inflammation, often progressing to necrotizing pneumonia, septic shock, and multiorgan failure, representing a challenge in medical practice due to its rapid progression and poor prognosis. We discuss a case of a 65-year-old diabetic male who developed severe CAP leading to septic shock, respiratory failure, and multiorgan dysfunction. Despite initial empirical antibiotic therapy, the patient deteriorated and died within 24 hours of ICU admission. Blood and sputum cultures later revealed methicillin-sensitive* S. aureus *(MSSA) positive for PVL. This case highlights the challenge of early diagnoses, the importance of prompt recognition, and the role of targeted therapies in infection control, antibiotics, and intravenous immunoglobulin. Early identification of PVL in suspected cases of severe pneumonia can improve survival, especially if treated with appropriate antibiotics and immune modulators within the first 24-48 hours. Although it is not a common diagnosis, clinicians should be aware of this possibility, especially if dealing with severe refractory shock, even in immunocompetent individuals.

## Introduction

*Staphylococcus aureus (S. aureus)* is responsible for approximately 2% of community-acquired pneumonia (CAP) cases, a significant but relatively rare contributor to pneumonia. Among the various virulence factors produced by this bacterium, one of the most concerning is Panton-Valentine leukocidin (PVL), a potent toxin primarily associated with severe skin and soft tissue infections (SSTIs). However, PVL is also a key factor in the development of necrotizing pneumonia, a life-threatening condition characterized by rapid lung tissue destruction. Although PVL is present in less than 2% of *S. aureus* isolates, its impact on the severity of infection, particularly in necrotizing pneumonia, is profound. The toxin works by forming pores in the membranes of neutrophils and macrophages, leading to cell lysis and apoptosis (programmed cell death), triggering the release of various inflammatory mediators that exacerbate the infection and contribute to tissue damage [1-3].

PVL-associated pneumonia typically affects previously healthy individuals, often beginning with a flu-like illness. This early prodrome is quickly followed by a rapid and severe progression of symptoms, including septic shock, respiratory failure, and systemic organ dysfunction. Patients often develop multilobar consolidation, pleural effusion, and airway hemorrhage. This rapid progression underscores the aggressive nature of PVL-driven pneumonia. The presence of certain clinical and laboratory findings, such as leukopenia, thrombocytopenia, and pleural effusion, are significant predictors of poor outcomes and important markers of infection severity [1,4].

Early diagnosis of necrotizing pneumonia due to PVL-producing *S. aureus* can be challenging, especially in young and healthy patients. The typically preceding flu-like syndrome, also indicating staphylococcal toxin production, can be confused with a simple non-specific viral illness. Other clinical and laboratory markers of this entity are hemoptysis, hypotension, leukopenia, and very high serum concentrations of C-reactive protein (CRP). Multilobar alveolar infiltrates are usually seen, and cavitations and pleural effusions commonly develop. 

The mortality rate associated with PVL-related pneumonia is notably high, with some reports indicating that it can be as high as 75% in severe cases [5]. Despite its relatively rare occurrence in the general population, PVL expression is considered a key marker for the potential severity of infection, regardless of whether the strain of *S. aureus* is methicillin-resistant (MRSA) or methicillin-sensitive (MSSA). The toxin's presence signals an increased likelihood of a rapidly worsening infection, which can quickly lead to organ failure and death if not treated appropriately. As such, early recognition and prompt anti-staphylococcal regimen and antitoxin agents like clindamycin, linezolid, and intravenous immunoglobulin (IVIG) are critical for improving patient outcomes and preventing fatal consequences. However, no clear risk factor that reliably predicts which patients will progress to severe disease has been identified, making clinical vigilance and prompt intervention paramount. Healthcare providers should maintain a high index of suspicion for PVL infections, particularly in patients presenting with severe pneumonia and rapidly escalating symptoms [6].

## Case presentation

The patient was a 65-year-old male with a history of diabetes who presented to the emergency department (ED) in April 2023 with a five-day history of fatigue, productive cough, and fever unresponsive to antipyretics. The patient denied other symptoms or recent travel. On admission, he was alert and oriented, febrile at 38.9 °C, with a blood pressure of 124/62 mmHg and a heart rate of 135 bpm. He exhibited signs of respiratory distress with a respiratory rate of 35 breaths per minute, accessory muscle use, and oxygen saturation (SpO_2_) of 60% despite receiving high-flow oxygen at 15 L/min via a non-rebreather mask. There were no clinical signs of fluid overload. Arterial blood gas analysis revealed hypoxemic respiratory failure with a partial pressure of oxygen (pO_2_) of 31 mmHg [normal range (NR): 83-108 mmHg] and elevated lactate levels at 6.8 mmol/L (NR: <1.3 mmol/L). The patient was promptly intubated and placed on mechanical ventilation. During sedation for intubation, he developed hemodynamic instability, requiring vasopressor support with norepinephrine.

**Figure 1:**
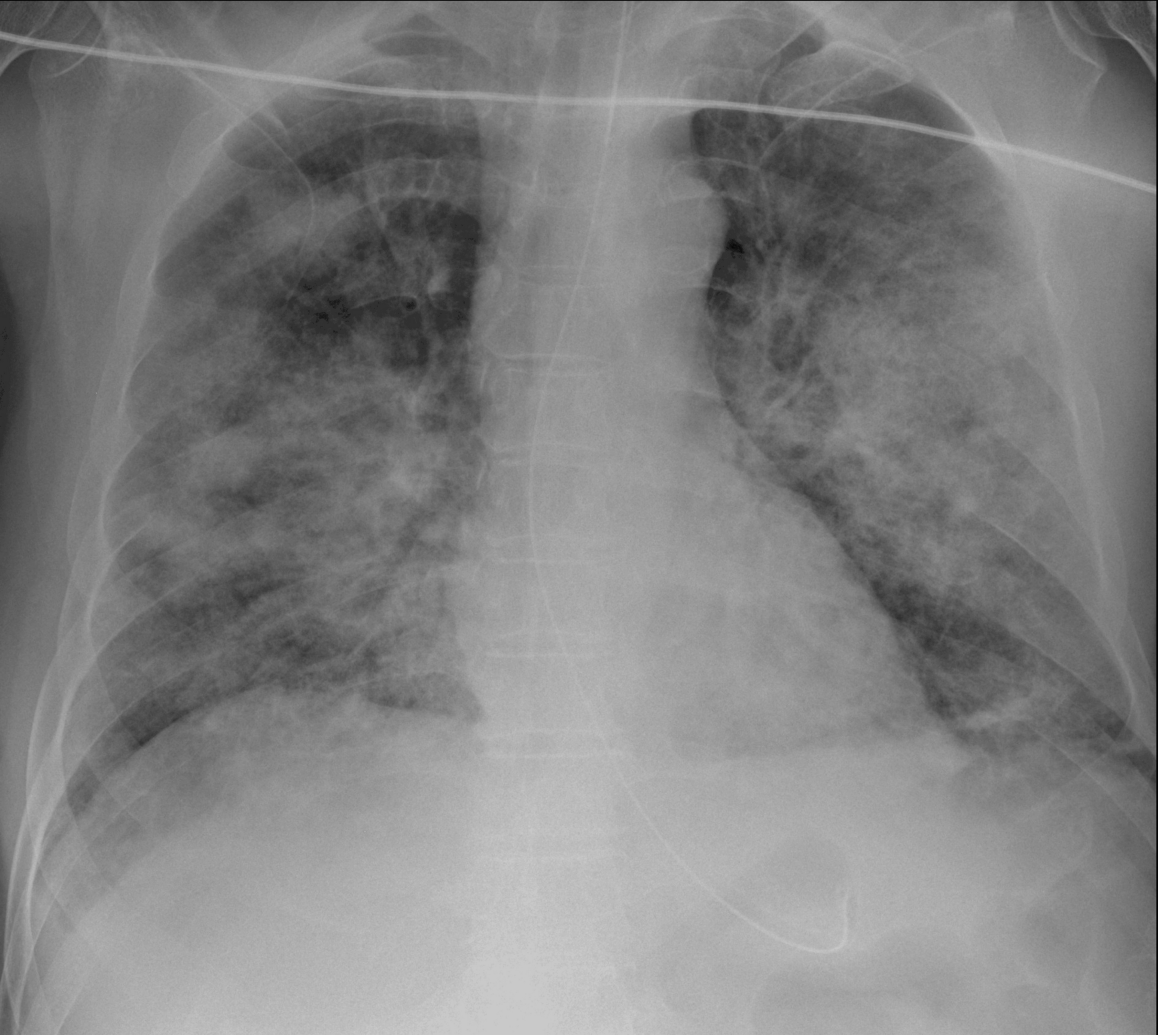
Chest X-ray at admission Chest X-ray at admission with severe diffuse bilateral infiltrates

**Figure 2:**
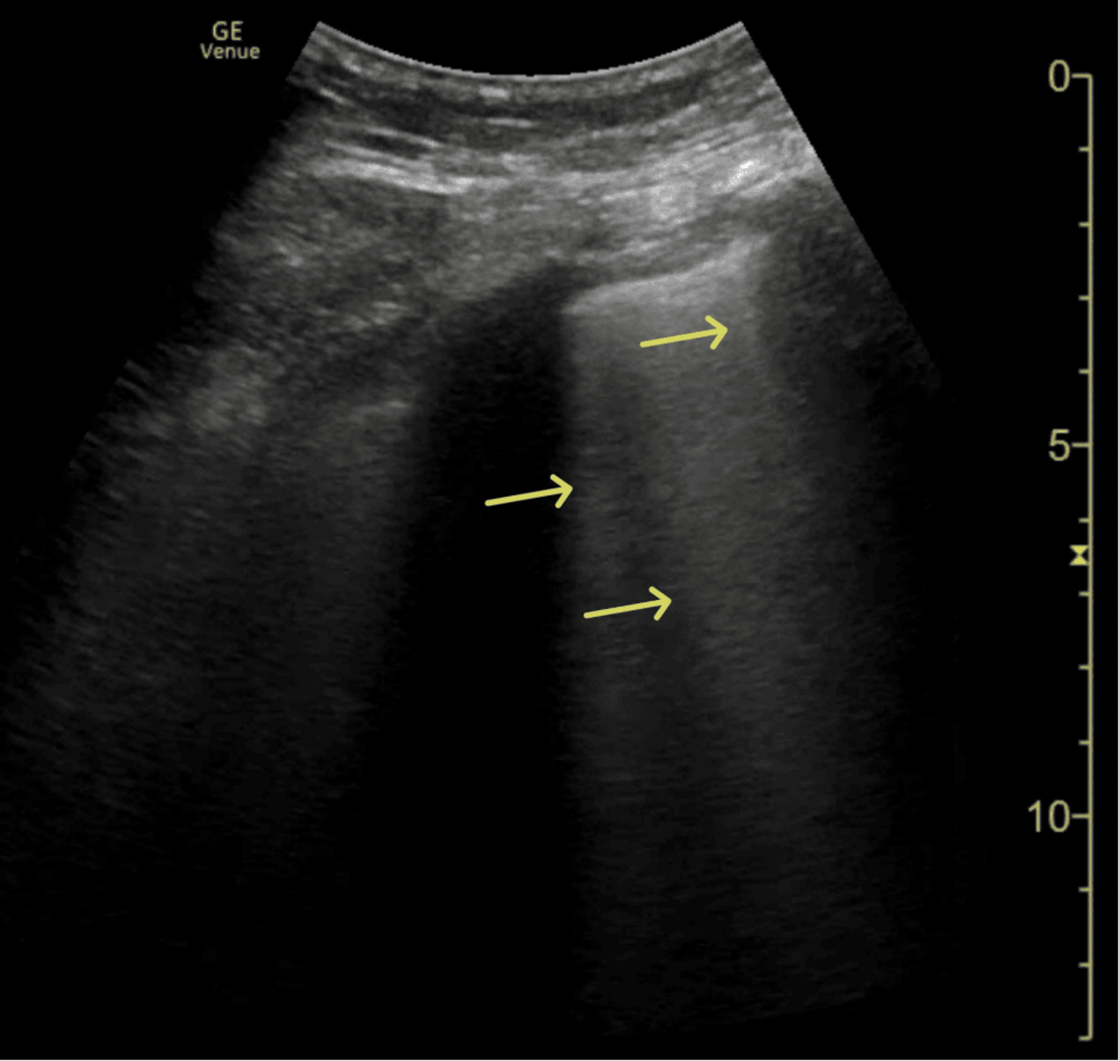
Point-of-care thoracic ultrasound Thoracic ultrasound showing bilateral B-lines (yellow arrows), without pleural effusion or subpleural consolidations

**Figure 3:**
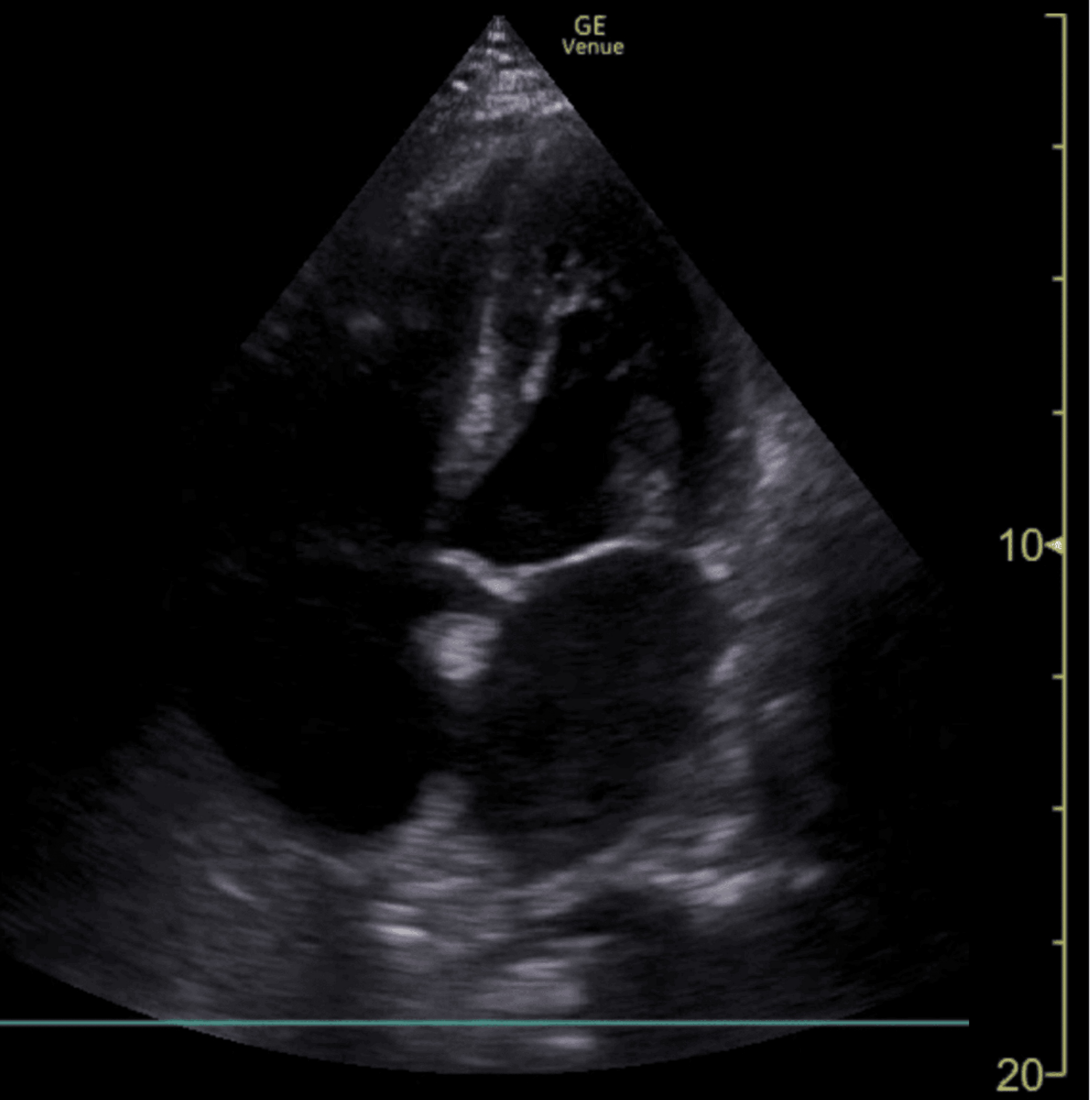
Transthoracic echocardiography in apical 4-chambers Transthoracic echocardiography in apical 4-chambers with preserved global left ventricular systolic function showing no evidence of enlargement of the right ventricle and no evidence of pericardial effusion

Initial laboratory findings (Table 1), demonstrated severe leukopenia, thrombocytopenia, acute renal failure, and an elevated inflammatory response with high levels of CRP and procalcitonin. A chest X-ray (Figure 1) revealed diffuse bilateral infiltrates. Point-of-care thoracic ultrasound showed bilateral B-lines without pleural effusion or subpleural consolidations (Figure 2). Transthoracic echocardiography (TTE) (Figure 3) revealed no myocardial dysfunction, with preserved global left ventricular systolic function, no evidence of right ventricular dysfunction, or signs of pulmonary embolism. Pericardial effusion was excluded.

**Table 1:** Laboratory results Initial laboratory findings showing severe leukopenia, thrombocytopenia, acute renal failure, and a severe inflammatory response with high levels of C-reactive protein and procalcitonin

Laboratory study	Results	Normal range
Leukocytes, x10^9^/L	1.40	3.70-9.50
Platelets, x10^9^/L	107	170-480
Serum creatinine, mg/dL	2.47	0.70-1.20
C-reactive protein, mg/dL	30.45	<0.50
Procalcitonin, ng/mL	>100	<0.10

A diagnosis of CAP complicated by acute respiratory distress syndrome (ARDS) was established. Respiratory viral panel testing, including influenza A/B and severe acute respiratory syndrome coronavirus 2 (SARS‑CoV‑2), was negative, as were urinary antigen tests for *Streptococcus pneumonia (*S. *pneumoniae) *and *Legionella pneumophila (L. pneumophila)*. In the absence of risk factors for MRSA or *Pseudomonas aeruginosa (P. aeruginosa)*, and considering local epidemiology, with a low and stable resistance rate of *S. pneumoniae* to penicillins and other β-lactams, empirical combination therapy with a β-lactam (amoxicillin-clavulanate) plus a macrolide (azithromycin) was initiated.

Despite aggressive management, including fluid resuscitation, vasopressor therapy with norepinephrine (>2 mcg/kg/min), vasopressin (0.03 IU/min), corticosteroids (hydrocortisone 200 mg/day), and renal replacement therapy, the patient's condition rapidly deteriorated, progressing to refractory shock. All efforts to optimize supportive care were unsuccessful, and the patient experienced a rapid clinical deterioration, culminating in death within 24 hours of intensive care admission. Blood and sputum cultures subsequently identified MSSA positivity for PVL.

## Discussion

While* S. aureus *is not a common cause of CAP, PVL-producing strains can result in fatal necrotizing pneumonia, which is characterized by fever, hemoptysis, and severe lung tissue destruction. The discordance between the clinical severity of the disease and the inflammatory markers often raises suspicion for PVL infections [2]. Necrotizing pneumonia caused by PVL-producing *S. aureus *is highly lethal, with a median survival of just four days [5]. Leukopenia and hemoptysis are common and critical indicators of PVL infection [4]. Histopathological examination typically shows necrosis, ulceration, and hemorrhage in the respiratory tract, which are indicative of the damage caused by the toxin [6]. The final diagnosis of PVL infections is confirmed through polymerase chain reaction (PCR), which can accurately identify PVL-producing strains of *S. aureus*, as conventional bacterial culture methods may fail to detect the toxin itself [3].

Treatment options for PVL infections generally involve surgical drainage of any abscesses, the combination of antibiotics particularly with antitoxin activity, and, in some cases, IVIG therapy. Selecting an effective antibiotic regimen is crucial for controlling the infection. While initial therapy typically includes broad-spectrum antibiotics, such as β-lactams or vancomycin, to cover the most common bacterial pathogens, adding clindamycin or linezolid can be a key component in treating PVL infections. Clindamycin works by inhibiting bacterial protein synthesis, thereby reducing the production of the PVL toxin. This helps limit tissue damage caused by the toxin and prevents further necrosis and systemic inflammation [1,6].

In cases of necrotizing pneumonia, where tissue necrosis impedes the penetration of antibiotics, clindamycin ensures that the therapeutic concentration of antibiotics reaches the infection site and neutralizes the effects of the PVL toxin. Linezolid, a synthetic oxazolidinone that suppresses protein synthesis and has enhanced efficacy against PVL-producing strains, is a good alternative to clindamycin. Linezolid is also a good choice as it has good penetration in the lungs and soft tissues [7]. Although evidence supporting the efficacy of IVIG in treating PVL infections remains limited, it may be considered in severe cases, especially if the disease is progressing rapidly or if the patient is at risk of toxic shock. High-dose regimens (2 g/kg as a single dose or 1 g/kg/day for two consecutive days) have been suggested, although the optimal dosing regimen is a subject of ongoing research [5].

Early recognition and targeted management, including the use of antitoxins and broad-spectrum antibiotics, can significantly improve survival rates. In cases of necrotizing pneumonia, where surgical drainage may not be possible in the early stages due to global lung necrosis, the infection may evolve into a more localized abscess or pleural effusion, which can be drained later [4]. Additionally, supportive care such as mechanical ventilation, fluid resuscitation, and vasopressor therapy should be employed to manage septic shock and multiorgan failure. However, even with aggressive treatment, the prognosis remains poor in many cases of PVL-associated necrotizing pneumonia, underlining the importance of early detection and targeted therapy [6].

In our case, the choice of antibiotics was made considering the absence of risk factors for MRSA or *P. aeruginosa* and local epidemiology. Amoxicillin-clavulanate is a broad-spectrum antibiotic and a good option for treating severe MSSA infections, even covering possible penicillin resistance. However, it does not have an antitoxin effect. The index of suspicion for necrotizing pneumonia due to PVL-positive *S. aureus* was low, as the typical features of infection with these strains, such as an influenza-like prodrome and hemoptysis, were absent. Additionally, the infection occurred outside the influenza season in Portugal, with negative influenza testing. However, the rapid clinical deterioration, leading to refractory shock and multiorgan dysfunction, could have triggered suspicion of toxin-producing strains and prompted the addition of an antibiotic with antitoxin activity. The addition of clindamycin or linezolid, along with immunoglobulin, could potentially have altered the clinical course. However, the severity at the time of presentation suggested a likely fatal outcome. 

Patients presenting with necrotizing community-acquired or hemorrhagic pneumonia should prompt consideration for PVL detection. A sample (e.g., pus, swab of exudate, sputum) should be sent to the local microbiology laboratory for culture and testing for the presence of PVL. The methods currently available to detect this toxin include PCR, enzyme-linked immunosorbent assay (ELISA), immunochromatographic test (ICT), lateral flow assay (LFA), and mass spectrometry (MALDI-TOF-MS). All these methods have high sensitivity and specificity, and can rapidly detect PVL, some within minutes [8]. This rapid toxin detection could play an important role in the prognosis, potentially avoiding fatal outcomes. Despite an initial low index of suspicion for PVL-related disease, toxin detection could change the clinical course, allowing physicians to add an antitoxin agent in time.

In Portugal, there are no specific strategies/guidelines for preventing this type of infection in the community, as it is extremely rare. However, PVL-*S. aureus* has been gaining relevance and represents a significant public health problem worldwide. Prevention strategies aimed to reduce the incidence of PVL-related infections, such as decolonization of patients with recurrent PVL-*S. aureus* infections, contact tracing, and monitoring adherence to decolonization strategies, could help minimize the impact of this condition. Clinicians should be aware of the rapid progression of PVL-associated pneumonia and the potential benefits of early intervention with appropriate antibiotics and immune modulators. Healthcare institutions should implement local protocols for severe CAP, particularly when signs of necrotizing pneumonia or multiorgan failure are present, emphasizing the importance of early administration of an antitoxin agent and immunoglobulin, even when antibiotic therapy has already been initiated. Furthermore, rapid detection of PVL in severe CAP cases could be a game-changer. Future research is needed to explore the potential benefits of a rapid bedside tool for detecting *S. aureus* toxins.

## Conclusions

This report highlights the devastating potential of PVL-associated infections in immunocompetent individuals. Early recognition and targeted therapies, including the use of clindamycin, linezolid, and IVIG, could improve patient outcomes. However, given the severity of PVL-related necrotizing pneumonia, the mortality remains high. This report serves to alert clinicians to the importance of considering PVL in severe CAP, thereby enabling the implementation of more effective treatment strategies in the crucial first 24-48 hours. Clinicians should be aware of the rapid progression of PVL-associated pneumonia and the potential benefits of early intervention with appropriate antibiotics and immune modulators.
